# Why publish? An interview study exploring patient innovators’ reasons for and experiences of scientific publishing

**DOI:** 10.1186/s40900-024-00589-9

**Published:** 2024-06-06

**Authors:** Marie Dahlberg, Jamie Linnea Luckhaus, Henna Hasson, Hanna Jansson, Madelen Lek, Carl Savage, Sara Riggare, Carolina Wannheden

**Affiliations:** 1 Department of Learning, Informatics, Management and Ethics, Medical Management Centre, Karolinska Institutet, Tomtebodavägen 18A, 171 77 Stockholm, Sweden; 2 Department of Women’s and Children’s Health, Participatory eHealth and Health Data, Uppsala University, Uppsala, Sweden; 3 Center for Epidemiology and Community Medicine (CES), Region Stockholm, Stockholm, Sweden; 4 Uppsala University Centre for Disability Studies, Uppsala, Sweden

**Keywords:** Patient author, Patient and public involvement, Patient-driven innovations, Patient agency

## Abstract

**Background:**

Scientific publications featuring patient-driven innovations (i.e., innovations that are developed and driven by patients or informal caregivers) are increasing. By understanding patient innovators’ experiences of research publication, the scientific community may be better prepared to support or partner with patient innovators. Thus, the aim of this study was to explore patient innovators’ reasons for and experiences of authoring scientific publications about their innovations.

**Methods:**

Qualitative semi-structured interviews were conducted with 15 international patient innovators from three continents who had published in scientific journals. Participants were identified through a scoping review on patient-driven innovations and snowball sampling. Interviews were conducted from June to October 2022 and the data was analyzed using the Framework Method.

**Findings:**

Participants’ reasons for publishing in scientific journals were to strengthen the roles and voices of patients and informal caregivers, and to get recognition for their innovations. Some published as a response to serendipitous opportunities. Several positive experiences were reported: collaborations defined by transparency, mutual respect, and meaningful participation; learning and competence development; and gained confidence regarding the value of lived experiences in research. Participants also reported negative experiences, such as cultural barriers manifested as conservatism in academia and power imbalances between participants and researchers, and structural barriers regarding academic affiliations and research funding.

**Conclusions:**

Despite progress in increasing patient and public involvement in research and publication, our study found that patient innovators still experience barriers. This suggests that continued efforts are needed to facilitate contributions from patient innovators and other public actors to the production of relevant and meaningful research.

**Supplementary Information:**

The online version contains supplementary material available at 10.1186/s40900-024-00589-9.

## Abstract (plain language summary)

Rapid technological advances over the past decades have resulted in many health innovations that enable persons living with chronic conditions to better manage their health conditions in self-care. Similarly, caregivers can provide more advanced informal care. Do-it-yourself health innovations, such as automatic insulin dosing for diabetes patients, have been developed by patients and informal caregivers whom we in this study defined as *patient innovators*. There is an increasing trend of research focusing on such patient-driven innovations. However, we know little about the driving forces of patient innovators to contribute to scientific publications about their innovations. Therefore, the aim of this study was to explore patient innovators’ reasons for publishing and their experiences thereof. We interviewed 15 international patient innovators who had experience of scientific publishing. Their main driving forces were to make patients’ voices heard and receive recognition for their innovations, which could facilitate spread to other patients. The patient innovators in our study had positive experiences and meaningful collaborations with researchers that contributed to developing their scientific skills. However, they also faced challenges, such as managing their health and professional occupations besides research, being questioned by peer reviewers, and difficulties tackling the research and publication system without academic affiliation or funding. Our findings suggest that despite strong driving forces and positive experiences of scientific publishing, patient innovators face barriers that need to be addressed to facilitate the publication process for contributors without academic experience.

**Supplementary Information:**

The online version contains supplementary material available at 10.1186/s40900-024-00589-9.

## Introduction

Rapid technological advances over the past decades have resulted in many disruptive health innovations that enable individuals to perform self-care tasks that have previously required healthcare’s resources and expertise [1]. The do-it-yourself open-source artificial pancreas system technology, developed by the #OpenAPS community, is an example of such an innovation that facilitates glucose control for persons living with diabetes type 1 [2]. Persons who experience unmet health needs (e.g., as a patient or informal caregiver) are important drivers in the development and spread of health innovations [3]. The Patient innovations website [4], which was developed to facilitate the spread of patient-driven innovations, listed over 1000 innovations in September 2023. Although online platforms and social media appear to be the main channels for patients and informal caregivers to exchange experiences and share their innovations, there is also an increase of peer-reviewed studies on patient-driven innovations [5].

An increasing number of scientific publications on patient-driven innovations are authored or co-authored by the innovators themselves [5]. The increase of patient-authored publications has been related to an increased interest from patients and informal caregivers to engage in research and publishing, as well as an increased recognition by major funders and the scientific community of patient participation in publications, editorial boards, publication steering committees, and at congresses [6]. There is a growing body of literature providing guidelines and recommendations to prepare patients and researchers to engage in collaborative research activities [7, 8]. This trend of putting patients’ experiences and competencies in the foreground is a sign of increased recognition of end-user perspectives and knowledge. Yet, how patients’ and public contributors’ experiences are taken into account in the construction of knowledge is not always clear [9]. The assumption underlying many strategies for patient and public involvement is that the research process is led by researchers, while the involvement of patients and public contributors may range from involvement as informants or study participants to co-authors [10]. However, there are several examples where patient innovators also take on a leading role as first authors of scientific publications [11–13].

Patient innovators, as defined in this study, are patients or informal caregivers who have developed and driven (i.e., lead the innovation process and spread) health innovations to address one or several unmet health needs [5]. Examples of unmet needs addressed by patient-driven innovations include self-care management, open sharing of information and knowledge, and patient agency in self-care and healthcare decisions [14]. Previous research suggests that autonomy is an important characteristic of patient innovators as they move from struggling with problems to developing solutions for their self-care and interaction with healthcare providers [15]. By developing and spreading innovations, patient innovators demonstrate empowering behaviors (e.g., sharing knowledge, supporting peers, and generating innovative ideas on individual and system levels) to influence self-care and healthcare [16].

Our previous research indicates that patient innovators have high ambitions reaching beyond research productivity, such as reaching societal impact by influencing clinical practice [17]. Yet, the motives that drive patient innovators to publish in peer-reviewed journals, as well as their experiences of publishing, merit further investigation. By increasing our understanding of how patient innovators reason about contributing to research production and their experiences of patient authorship, the scientific community may be better prepared to support or partner with patient innovators in research, supporting high-quality studies and publications. Thus, the aim of this study was to explore patient innovators’ reasons for and experiences of authoring scientific publications about their innovations.

## Methods

### Study design

We employed a qualitative research design based on semi-structured interviews, analyzed using the Framework Method [18]. The Framework Method can be described as a combination of an inductive and deductive approach to thematic analysis, whereby researchers first inductively develop an analytical framework that is thereafter applied deductively to summarize and organize data into a highly structured format. The clear and step-by-step process is particularly suitable for interdisciplinary collaboration. The study design and reporting were guided by the Guidance for Reporting Involvement of Patients and the Public, Short Form (GRIPP2-SF) [19] and the Consolidated criteria for reporting qualitative research (COREQ) [20].

### Participants

Inclusion criteria were that patient innovators had 1) developed and driven a health innovation based on their experience as a patient or informal caregiver, and 2) (co-)authored at least one publication about their innovation in a peer-reviewed journal. Participants were primarily recruited based on a scoping review of patient-driven innovations published by members of our research group [5]. We identified 37 patient innovators in the studies included in the scoping review. JLL contacted 28 patient innovators for whom we were able to find contact details through email, LinkedIn, ResearchGate, or via corresponding authors. Nine responded and consented to be interviewed. Six additional participants were recruited through snowball sampling [21], resulting in a total of 15 participants. The participants had developed various types of innovations: digital platforms (*n* = 4), mobile applications (*n* = 3), social innovations (*n* = 2), and technical devices (*n* = 6). The innovations were developed for various chronic conditions (autoimmune diseases, diabetes, digestive diseases, disabilities, hematologic disorders, neurological conditions, rare diseases, and allergies) and were published in diagnosis specific journals, multidisciplinary journals, and journals focusing on formative health research, healthcare digitalization, technology, and informatics. Participant characteristics and their publishing experiences are specified in Table 1.

**Table 1:** Participant characteristics (*N* = 15)

Characteristics	*N* = 15 (%)
**Gender**	
Women	8 (53)
Men	7 (47)
**Innovator role**	
Patient	11 (73)
Informal caregiver	4 (27)
**Residence**	
Europe	8 (53)
Middle East	1 (7)
North America	6 (40)
**Authorship experience**	
First or single author experience	10 (67)
Publications on other^a^ topics	5 (33)
**Total number of publications**^**b**^	
1 publication	2 (13)
2–5 publications	7 (47)
6–9 publications	3 (20)
≥ 10 publications	3 (20)

^a^Other topics than their patient-driven innovations

^b^Retrieved from Web of Science on May 31^st^, 2022

### Data collection

Data collection spanned June to October 2022. A collaborative team of researchers and patient innovators co-created a semi-structured interview guide focusing on the innovation journey, reasons for publishing, and experiences of the research and publication process (Appendix 1). The interview guide was collaboratively developed by the authors and patient innovators from our research team, except SR (who was also an interview participant). JLL, a native Swedish-American speaker trained in qualitative research, conducted the interviews online (via Zoom), in English, with a duration of 31–74 (mean 50) minutes.

### Data analysis

Data were analyzed in seven iterative steps. First, JLL recorded and transcribed the interviews verbatim using MS Office Transcribe [22]. The auto-generated transcripts were manually corrected. Second, MD listened to the recordings, whereafter MD and JLL read and re-read the transcripts to familiarize with the data. Third, MD and JLL independently open-coded three interviews by underlining interesting segments and labeling them with codes. Fourth, MD and JLL discussed the codes with CW and SR and created an analytical framework (i.e., a set of codes grouped into categories). MD and JLL tested the framework on three additional interviews, followed by an iterative process of refinements, which resulted in a framework consisting of thirty-seven codes with brief descriptions, grouped into seventeen categories (Appendix 2). Fifth, all transcripts were imported into the qualitative analysis software NVivo 12 [23]. MD and JLL divided the transcripts (including the initial three) between themselves and coded them independently using the framework (represented as nodes and sub-nodes in NVivo). They iteratively cross-checked the coding with each other and made minor refinements to the framework. Sixth, MD and JLL charted the data into a matrix in a Microsoft Excel spreadsheet [24], summarizing interviews (in rows) by categories (in columns), with verbatim words underlined (see illustrative example in Appendix 3). Seventh, themes and sub-themes were generated by reviewing the matrix and connecting data within and between interviews and categories, guided by the original research objectives. The generated themes and sub-themes were discussed and refined iteratively among all authors. Finally, illustrative quotations were selected; we made minor linguistic corrections to enhance readability [25].

### Patient and public involvement

This study was performed within the research program titled *Patients in the Driver’s Seat*, in which researchers and patient innovators collaborate as partners in research. One of the co-authors (SR) is a patient researcher and patient innovator. SR contributed to all stages of the research process, except the creation of the interview guide, and was also a study participant. To reduce the risk of bias in the analysis, SR did not have access to raw data transcripts; she was only involved in discussions of abstracted data (i.e., codes, categories, themes).

## Findings

**Fig. 1:**
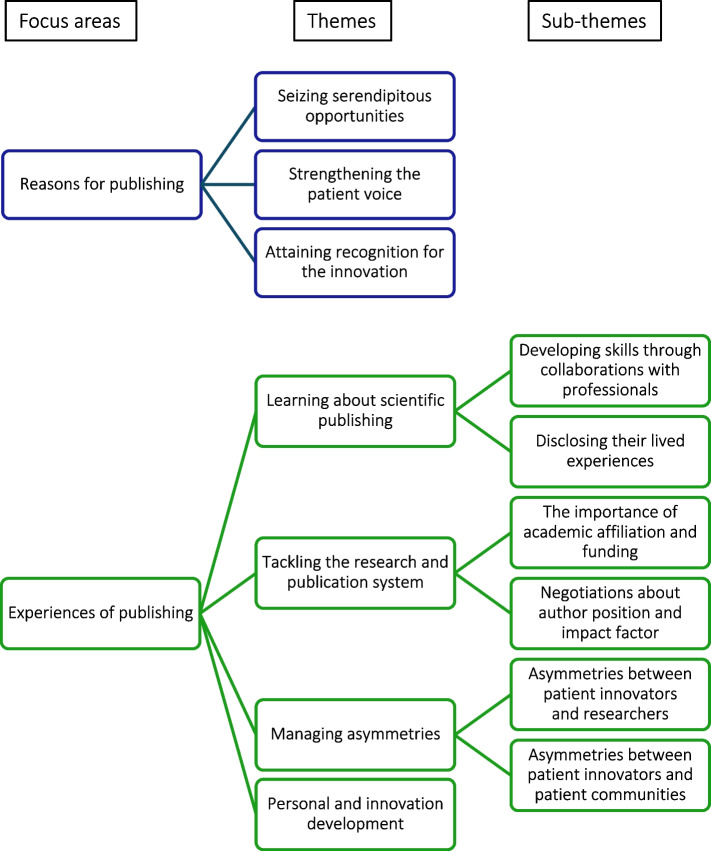
Focus areas, themes and sub-themes

We generated three themes reflecting participants’ *reasons for publishing* and four themes with six sub-themes reflecting their *experiences of publishing* (Fig. 1).

### Reasons for publishing

#### Seizing serendipitous opportunities

Our findings indicate that participants wanted to share their innovations in a scientific environment. However, the reasons for publishing in scientific journals were sometimes a result of serendipitous opportunities rather than a plan or ambition. Several of them had presented their innovations at conferences, which for some had led to new collaborations with researchers and one participant described being encouraged by a journal editor to publish. Participants referred to these opportunities as a combination of “luck factors” (#1) and being in the right place at the right time. When seizing serendipitous opportunities, participants described that they may not at first have understood the benefit of publishing. However, once their work was published, some got motivated to publish again.

*So, in the first publications my role was bringing in the patient voice and the patient perspective and the publications were often [about] co-production, patient reported outcome measures, and those types of things, where it is very much around understanding the patient’s perspective, so I was very happy about those invitations and gladly accepted… so that was how it started. (#4)*

#### Strengthening the patient voice

Participants described how they wanted to strengthen the voices of patients and informal caregivers in scientific publications. As one participant emphasized, “some of the best stuff in the diabetes community has come out of people with lived experiences” (#1). Another participant highlighted how patient-authored publications brought critical attention to specific communities, such as persons living with rare diseases. By sharing self-collected data concerning their health conditions, participants believed that they could contribute to an increased production and application of real-world evidence. Thus, they hoped that their publications could also inspire others to get involved in research and publishing. They emphasized that more voices from the patient community needed to be heard.

*Like, don’t just take what I say and run with it. Go and get some other perspectives as well. Because this will hopefully improve the quality of your output because you’ve got multiple points of reference… I don’t want to represent the [whole patient] community. (#10)*

Well-functioning collaborations with researchers was a critical factor for participants’ willingness to become involved in research. Involvement was seen as problematic in cases where it merely served to “tick a box” (#10). Participating on equitable terms throughout the process and being able to influence the study design was therefore experienced as imperative.

*If we can’t influence our part of defining the studies up front, we’re not interested…. Like we want to be able to influence studies in a direction to where we get something out of it. We don’t just want to be suppliers of instrumentation and self-tracking methods. There needs to be something in it for us where we can also inject some of our outstanding questions into the study designs. So, we can have some of our standing questions answered by participating in this study. So… we’re thinking about how we get something out of it. (#14)*

Although some participants perceived a gradual shift towards acknowledging the value of involving patients as partners in research, terms like “paradigm shift” (#5) and “a movement” (#6) were used to highlight the importance of further embracing the “growing network of patient researchers or patients that publish scientifically” (#6). One of the participants explained that “what we’re doing is culture change… we’re flattening a hierarchy of how research [is done]” (#5). Another participant urged healthcare and scientific communities to “acknowledge and be part of the conversation” (#10).

#### Attaining recognition for the innovation

Participants described that they used social media platforms, websites, newsletters, and word-of-mouth to disseminate and market their innovations in patient communities. They believed that while scientific publications are not the prime communication channel to reach patient communities, scientific publishing was considered the most effective way to gain attention and recognition among other key stakeholders (e.g., healthcare and academia, pharmaceutical companies, insurance companies).

*Publications are the space where [scientists and companies] get their information. So, it was really important to be able to reach people where they are… sort of entering their world… where they learn, and they gather information. (#11)*

Because scientific publishing was perceived to contribute to building awareness about patient-driven innovations, their purposes and progress, participants reasoned that it would bring credibility to their innovations that would eventually benefit other patients. In addition, some participants described how their publications had helped other patients in their dialogue with healthcare professionals, which reinforced their motivation to publish.

### Experiences of publishing

#### Learning about scientific publishing

Publishing was described as a learning process, which included developing hands-on academic writing skills, as well as developing the ability to assess how and when to disclose lived experiences in publications.

##### Developing skills through collaboration with professionals

Participants revealed how they initially struggled with understanding and adapting to what they described as the “mystery” of research. A particular challenge was learning how to write in a manner acceptable to the scientific community, while simultaneously adhering to the patient narrative.

*[I had to] disguise [the manuscript] in words and terms that the system recognizes. It’s kind of like a trojan horse, because if I can show that I know the system, it’s better. (#6)*

Learning to use academic language was identified as a key skillset and participants suggested that education and training could be valuable for inexperienced authors. Beyond writing skills, participants suggested that such training could encompass the different steps in the publication process (e.g., peer-review). As one of the participants declared: “it took years to recognize the magic and politics behind how to write a journal article*”* (#15). Apart from formal training, participants experienced that collaborating with researchers provided valuable support in the writing and publication process.

*I was so grateful that [the researcher] partnered with us and sort of guided us through the process because there’s so much to know about what publications to target, how you like set up a manuscript like how you should frame it to be most compelling… Not being part of the academic research community, we just never would have known that. So… I think [our research partner] helped us immensely in showing us the ropes. (#11)*

##### Disclosing their lived experience

Participants shared that conveying the patient perspective was their main priority in publishing. The specific objectives of the publications guided participants in deciding how much detail regarding their own lived experiences to disclose. If the purpose was to report on lived experiences of patients or informal caregivers, describing their patient or informal caregiver status could contribute to enhanced credibility of the publication. However, in epidemiological or clinical research, revealing their status was experienced as adding less value.

*So, in the first publication it was very personal... The second publication was not at all personal... I mean honestly, I think that for the second piece my role as a member of the community enabled us to develop a better survey (laughter). ‘Cause I live this disease every minute of my life and think it helped us to write better questions and know what information to collect... For the first one, you know... I felt like I had a platform to shine a light, so that sort of took precedence… from [a] patient status and then the other I felt like it was helpful, but it wasn’t a key part of the publication at all. (#11)*

Participants felt that the requirement by some journals to report academic titles could be a challenge. When reporting their role as a patient innovator, some received mixed reactions from the scientific community, ranging from appreciation to instances where their credibility was called into question. The peer-review process could work both for and against patient innovators’ favor. Participants believed that some reviewers judged patient innovators harder than researchers with academic titles, while they believed that others found it difficult to reject patient-authored papers even if they found them to be of questionable quality.

#### Tackling the research and publication system

Participants experienced obstacles in the research and publication system, including obstacles related to academic affiliations and funding, as well as negotiations about author position and journal impact factors.

##### The importance of academic affiliation and funding

Participants described how they learned about the importance of academic affiliations to pursue research and stressed an urgent need for “democratizing research” (#7) by making it easier for people from outside the research community to publish and access published data free of charge. Ensuring open access requires those who publish to pay article publishing charges, which can be difficult to fund without research grants, which in turn may require an academic affiliation. Further, participants expressed that accessing publications behind paywalls could be facilitated through an academic affiliation since many academic institutions have agreements with publishers for access.

*The organization we’re working with on this grant has article processing charges waived for these journals so we can publish open source if we do it in one of these. If we [publish] in these other [journals], then we either have to have it paywalled or we have to pay out of the grant…. Like there’s all these nuances of the business model of academic publishing and how that interacts with Open Access versus closed, paywalled. Some of the stuff that we are doing needs to be Open Access so that patients can see. (#8)*

##### Negotiations about author position and impact factor

Participants were surprised by the level of competition in research, which according to one participant ”hijacks all of academia into a status game” (#8). For example, participants described how negotiations about journal impact factors and author position distracted from the actual work and prolonged the publication process, which they feared could discourage some non-academics from contributing to publishing in scientific journals.

*It took a long time to get it published, because in the beginning everybody wanted to be number one and four…. And then we got to the point to write it up and there were too many people who wanted to be one and four (laughter). So, it just got dropped for a while until this one got tenure, and this one gave it to a colleague to write instead… and then, you know, to find the right journal and then to go through the process we have to keep redoing… and this and that. They put us through the mill. I can’t believe it actually got published. (#3)*

#### Managing asymmetries

Participants shared experiences of managing asymmetries between themselves and researchers, as well as patient communities.

##### Asymmetries between patient innovators and researchers

Participants perceived that they as patient innovators had experiences and expertise about living with a certain health condition or providing informal care that were complementary to the scientific knowledge and skills of researchers. To successfully contribute with their perspectives, participants emphasized the importance of transparency, mutual respect, and active participation in their collaborations with researchers. Some shared positive experiences of how researchers had encouraged and supported them to contribute:

*[The research team] was really open and willing to integrate our point of view…. What has worked well is with the way we were able to provide our feedback and provide our expertise [and] experience within the publication. The fact that we were encouraged and supported to be part of the scientific process... I’m thinking about publishing again. (#13)*

Participants also had negative experiences of perceived asymmetries between themselves and researchers. For example, one of the participants described how their lack of research training could lead to situations where they had to fight a “battle for legitimacy” (#14) to be acknowledged as co-authors of scientific publications. Another participant described publications as the “currency of the scientific community” (#11), emphasizing the importance of a publication record to be acknowledged among scientists. Further, participants described that their possibilities to engage full-time in a publication process depended on several factors that may differ from researchers’ possibilities. One factor was that living with their chronic health condition or providing informal care could sometimes limit their ability and energy to engage in the research and publication process. Another factor was that patient innovators may have other professional occupations to manage besides engaging in research and publication. Additionally, they experienced that in comparison to researchers who may be supported by research grants and resources offered by a university (e.g., coverage of open access publishing fees or access to publications behind paywalls), publishing was difficult for patient innovators to fund.

##### Asymmetries between patient innovators and patient communities

Participants also experienced asymmetries between themselves and the patient communities they strove to give voice to. For example, they believed that their experiences of making their voices heard distinguished them from most patients and informal caregivers. Participants further clarified that not everyone is privileged enough or has the time, skills, and will to be able to do unfunded work. Participants stressed that in their combined roles as patients or informal caregivers, innovators, and researchers, they had an obligation to represent their own needs as well as the needs of the patient and informal caregiver communities they identified with.

*I think that within patient-led innovation or patient [involvement in] publication we need to make sure that [we] reflect not only the voice of the educated ones and the knowledgeable ones, but also reflect the voice of the ones who do not have a voice. (#13)*

#### Personal and innovation development

Participants experienced that their engagement in research and publication could lead to both personal development and the development of their innovations. Some thought that what they had learned from publishing, for example regarding methodology and data collection, fostered procedures within their teams that helped the development of the innovation. Participants also felt that being evaluated in research collaborations and by reviewers in the publication processes had helped them to mature professionally and engage in more research. A participant described this development in terms of building an “evidence pyramid” (#1) that evolved from the research that led to publications, which enabled more research resulting in additional publications. Publications could also lead to new connections and networks (e.g., with industry, regulatory agencies, or other researchers or patient innovators), which for some paved the path for new collaborations.

*The learning comes before [publishing], especially collecting the data, doing the interviews with the participants… and then we feed [the results] into the development of technology and the method. So that’s like two parts, getting the data from those collaborations… and then of course like marketing the instrument by having articles out there…. If we’re reaching out to potential collaborators, then we can reference these articles…. (#14)*

## Discussion

This study explored patient innovators’ reasons for and experiences of publishing about their health innovations in scientific journals. Reasons included serendipitous opportunities, strengthening the roles and voices of patients and informal caregivers, and attaining recognition for the innovation to enhance dissemination. Patient innovators reported positive experiences of publishing, such as competence development and gained confidence regarding the value of incorporating patient experiences in research. Barriers that complicated publication were mainly related to experiences of a conservative research culture, manifested as asymmetries between patient innovators and researchers, and structural barriers such as lacking academic affiliations and research funding. Literature specifically targeting patient innovators as researchers and authors is still scarce [5]. However, we found that the experiences of patient innovators in our study share similarities to experiences of patient authors more generally (i.e., patient authors who do not also identify as patient innovators). Thus, we will discuss our findings in relation to literature on patient involvement in research and publishing.

### Research collaborations facilitate publishing

Our findings indicate that the research collaborations could facilitate publishing for patient innovators. In previous research, it has been argued that to achieve meaningful research collaborations involving patients, researchers need to understand and embrace the importance and benefits of including patients as partners [26]. Further, the importance of consistent and continuous collaboration throughout the research process has been emphasized [27]. In our study, patient innovators emphasized collaborations defined by transparency, mutual respect, and fair participation throughout the research process. Supportive and encouraging researchers were perceived to help patient innovators to advance their scientific skills. The facilitating conditions that participants highlighted share similarities with guiding principles of participatory design processes (e.g., democracy, mutual learning, and collective creativity) [28]. This suggests that participatory design principles may be applicable to guide collaborative publication processes.

### Conservative research culture challenges publishing

Patient innovators in our study engaged in scientific publishing as a strategic step to reach key stakeholders. However, they described the struggle to understand the research system and to become recognized as co-authors. For example, participants debated the requirement of academic titles, when and how much detail to share about their background, and how to balance their narrative while adapting to an academic language for acceptance in the scientific community. These experiences suggest that patient innovators meet various obstacles in contributing to knowledge production. Patient innovators’ experiences are in line with previous research highlighting that the contribution of patients’ lived experiences is still not fully acknowledged in the research community [26]. For example, patient innovators perceived a risk of being exposed to peer review bias, which concerns deviations from objective evaluations of research findings [29]. Specifically, they feared that their work was evaluated based on opinions about them as patients and informal caregivers (i.e., *ad hominem bias* [30]), rather than based on the quality of their work. Peer-review practices in research have been described as conservative, disfavoring research that is “paradigm-shifting”, “revolutionary”, and “frontier” [31], which may apply to papers authored by patient innovators and other patient and public contributors. Clarifying the contributions of all co-authors may reduce the risk of bias against specific persons or roles. This could be facilitated by introducing standard affiliation terms for authors with lived experiences as patients or informal caregivers [32]. This could also contribute to democratizing research, as was emphasized by participants of our study. Other measures that have been recommended to address the conservative research culture are to include patients and the public on review boards [32], as well as in research funding committees [33]. Education for editors-in-chief and the broader scientific community has also been suggested to help circumvent misconceptions and antagonistic attitudes regarding patient participation [34].

A conservative research culture was experienced within collaborations where patient innovators perceived that they merely served to “tick a box”. They wanted to be involved in the entire research process to ensure relevance in regard to their innovation, and to contribute with their innovation competence and lived experiences to the research. This is in line with research recommending that patients be involved before, during, and after publications to minimize asymmetries and maximize quality of research collaborations and publications [7]. However, research legislation and regulations confuse patients and researchers alike on how to best involve patients [26]. Although an increasing trend of patient-authored publications during the past decade, they compose only a fraction of all scientific literature [6, 35, 36]. Notably, publications capturing patients’ perspectives, experiences, priorities, and needs reached over 16,000 in 2022, yet only 29 of these included authors with actual patient experience [36]. This suggests that even though patient and public involvement in research is increasingly encouraged, few are involved as co-authors.

Structural challenges for publishing that patient innovators raised concerned the perceived advantage of academic affiliations and the high costs related to research and publishing. Although researchers and editors have argued that patients should be able to share their unique competences and perspectives without academic affiliations [34], patient innovators in this study experienced that collaborations with affiliated researchers or research organizations were necessary to access academic resources and to secure funding for research and publication costs. Therefore, patient innovators stressed the importance of efforts to enable non-academics to engage in research and publication. Examples of such efforts are the requirements of declarations of patient and public involvement that are increasing among funding organizations [37, 38] and scientific journals (e.g., British Medical Journal), as well as various frameworks that have been developed for supporting and evaluating patient and public involvement in research [10]. In addition, to increase public accessibility to research, a growing number of journals (e.g., Research Involvement and Engagement) and stakeholders recommend or require plain language summaries [39].

### Limitations

There are certain limitations to consider when interpreting the trustworthiness of our findings and assessing their transferability to a larger population of patient innovators. Our study was limited to individuals who we identified as patient innovators with experience of scientific publishing. Identifying scientific articles co-authored by patient innovators can be difficult as author roles are not always well-described [5]. Thus, although we were able to identify and include a heterogeneous sample of patient innovators residing in various countries and representing a variation of chronic conditions and types of innovations, our sample may not be representative of all patient innovators who have experiences of scientific publishing. Participants’ personal characteristics, their desire to disseminate their innovations in scientific journals, and their opportunities to participate in scientific publishing may stand out from the larger population of patient innovators. Further, individuals with lived experiences as patients or informal caregivers who have authored scientific publications but do not identify as patient innovators (e.g., individuals who identify as patient authors or patient researchers) may have different driving forces and experiences of publishing than reported in this study. We did not ask participants about sociodemographic factors (e.g., age, education level, professional status), the years of experience with scientific publishing, and infrastructure (e.g., systems and tools) available to them for publishing, which limits the transferability of our findings.

## Conclusions

Patient innovators engaged in scientific publishing to strengthen the patient voice and to get recognition for their innovation. They wanted to contribute with their lived experiences as well as with their innovation competence to improve research and health outcomes. Although patient innovators had positive experiences of research and publication processes, they faced cultural and structural barriers. Our findings suggest that continued efforts are needed to facilitate for patient innovators, as well as other patients and members of the public, to contribute with their experiences and expertise to the production of relevant and meaningful research.

### Supplementary Information

Supplementary Material 1.

Supplementary Material 2.

Supplementary Material 3.

Supplementary Material 4.

## Data Availability

No datasets were generated or analysed during the current study.
