# Supplementary material for: Why publish? An interview study exploring patient innovators’ reasons for and experiences of scientific publishing
Source: Res Involv Engagem. 2024 Jun 6;10:54. doi: 10.1186/s40900-024-00589-9 (PMC11157806; doi:10.1186/s40900-024-00589-9)
Supplement: Supplementary file 1 — Supplementary Material 1. [file 40900_2024_589_MOESM1_ESM.pdf]

Appendix 1: Interview guide

The journey of the innovation (including development, implementation, scale up)

1. How would you describe your innovation in just 2-3 sentences?
    a. What was the unmet need (or needs) that your innovation addressed?

Purpose/effects of publication

2. What led you to decide to write a scientific publication?
    - Did you have any specific goals/aims?
    - What effects/impact were you aiming/hoping for?
3. Have your aspired effects/impacts been fulfilled? How?
4. Has your publication led to other effects or reactions that you did not aspire/foresee?
    - Tell me more…
    - Positive? Negative?
    - Did the research in anyway become a barrier to your innovation thriving?
    - For you? (legitimacy, credibility, …)
    - For other patients/caregivers? (self-care)
    - For healthcare? (e.g., changed processes/services)
    - For the innovation? (e.g., spread of the innovation, adaptation of innovation)
5. How have your scientific publications impacted the continuous development and spread of your innovation? (everything from design, developing, testing, implementing, etc.)

Publication process and distribution of roles/responsibilities/power

6. At what stage in your innovation journey did you write your (first) scientific publication about the innovation?
    - How was the publication process initiated? How did it start?
7. How would you describe your role and responsibilities in the publication process?
    - What roles/responsibilities did your co-authors have?
    - Was it clear to you what was expected from you?
    - Did you feel that you were involved/able to influence the work to the extent that you desired?
8. What is your overall experience of the research and publication process?
    - What was it like to work with the project and/or article?
    - Do you have previous experience of research and scientific publishing or was this your first time?
    - What are your experiences of working with traditional/established researchers?
9. Who took the initiative to write the publication?
    - How would you describe your role and responsibilities?
        - Was it clear to you what others expected from you?
    - How would you describe the roles and responsibilities of others?
        - Was it clear what you could expect from others?
    - Who led the overall work with the publication? Why?
        - Did you feel that you were involved/able to influence the work to the extent that you desired?

10. If I would ask you to reason about the power distribution between you and your co-authors in this publication, what are your reflections?
    - Did you feel that power was equally distributed?
        - How does the scientific process hinder/promote equitable distribution of power and knowledge between stakeholders?
    - Did you feel empowered to influence the process as much as you wanted?
    - What empowered you/your stakeholders?

On being open about your experiences of being a patient/informal caregiver

11. In your publication(s), what were your thoughts about explicitly mentioning your experience as a patient/informal caregiver?
    - Do you think that this is important for others to know? Why/why not?
    - Have you received any reactions on mentioning your experience in publications?

To finalize…

12. If you think about your experiences of scientific publishing:
    - What has worked well?
    - What would you like to be different in the future?
13. Is there anything else you would like to share that we have not talked about?
14. Do you know of any other patient innovators who have published that we may contact?
