# Supplementary material for: Why publish? An interview study exploring patient innovators’ reasons for and experiences of scientific publishing
Source: Res Involv Engagem. 2024 Jun 6;10:54. doi: 10.1186/s40900-024-00589-9 (PMC11157806; doi:10.1186/s40900-024-00589-9)
Supplement: Supplementary file 2 — Supplementary Material 2. [file 40900_2024_589_MOESM2_ESM.pdf]

Appendix 2: Analytical framework

Categories	Codes with definitions
1. Seized opportunity	1. Invited to publish: Innovator was invited to publish or participate in publication or initiated it themselves<br>2. Seize opportunity: Seize an opportunity to do something many other patients do not get to do
2. Change-making	3. Informed health services and research: Ideas on how to generate evidence to highlight the needs of patient,caregiver population in health services and research (through the innovation and publication)<br>4. Real world research and evidence: Desire or need to challenge traditional research (RCTs and clinical data) to generate also non-clinical specific data (real-world research and evidence)<br>5. Targeting healthcare/research:Publishing to create and contribute to paradigm shift towards increased and improved patient participation in healthcare and research<br>6. Targeting patients/healthcare/research: publishing to share patient perspective (knowledge and experiences) make data available, and contribute to learning and advance understanding of condition or daily experiences<br>7. Targeting patients: Inspiring other patients to take action (use of innovation, research, publishing)<br>8. Translation into practice: Ideas about how to translate innovation into knowledge and changes in practice (e.g., integrated care)
3. Recognition	9. Audience: Views, attitudes, ideas, experiences about the audience that is targeted or not targeted, reached or not reached through scientific publishing<br>10. Marketing of innovation: Publication to demonstrate or show the value and work of the innovation<br>11. Recognition: Publishing to bring recognition to innovation and innovator
4. Access and ownership	12. Access: Publishing or accessing requires someone to pay<br>13. Ownership: Journal embargo deprives innovator of their own story
5. Attitudes and culture	14. Closed community: Scientific journals and/or communities experienced as a closed community for academics; risk of missing valuable experiences shared by patients<br>15. Rigid healthcare and research practices: Challenging traditional healthcare and research practices miss or risk missing the target
6. Authorship	16. Authorship role: Experience of authorship role (positive or negative)(level of participation)<br>17. Publishing identity: Publishing one's identity (as patient or informal caregiver) to make patient perspective available<br>18. Writing process: Experience of the actual writing process (easy or difficult)(postivie or negative)(level of participation)

7. Collaboration	19. Ideas or experiences regarding the value of collaboration between disciplines
8. Competence	20. Competence-evaluated: Experiences of how own competence and innovation being judged or evaluated (positive or negative)<br>21. Own-competence: Experiences of the research process and own competence to conduct research (e.g., analyze and synthesize data)
9. Guidance	22. Experience of guidance during the research and writing process (positive or negative)(level of participation)
10. Power dynamics	23. Conditions for participation: Conditions that impact level and ability to participate in research and publication process<br>24. Information asymmetry: Patients and academics have access to different (complementary) information and also use different language; in other words, academics cannot capture the full richness of patient suffering, related to the lived experience<br>25. Role of patient: Traditionally, academia conducts research on rather than with patients (yes or no).
11. Time-frame	26. Time frame: Experiences of tempo of research or publication process and getting hungup on steps such as ethical approval
12. Attention	27. Attention: Publishing brings attention to one's work, the innovator, or innovation<br>28. Dissemination: Publishing contributes to dissemination of innovation or publication<br>29. Reactions: Reactions to publication (positive or negative) (little or a lot of attention)
13. Connections	30. Publishing contributes to build new connections with other patients and, or research team and, or funders
14. Credibility	31. Funding affordabilty: Publishing brings credibility that enables funding and users' affordability (e.g., insurance)<br>32. Innovation credibility: Publishing brings credibility to innovation and research topic(s)<br>33. Innovator credibility: Publishing brings credibility because innovator is identified as a "researcher" (which can create mixed feelings)<br>34. Perceived value: How innovator perceives the value of their own publication
15. Innovation	35. Developments: Publishing led to developments of the innovation (e.g., methods or data collection, work processes), yes or no
16. Inspiration	36. Patient perspective contributes to inspire or open minds of people (e.g., clinicians
17. Paradigm shift	37. Fair patient participation throughout the research process identified as a new research paradigm and viewed as valuable and empowering
