# Supplementary material for: Why publish? An interview study exploring patient innovators’ reasons for and experiences of scientific publishing
Source: Res Involv Engagem. 2024 Jun 6;10:54. doi: 10.1186/s40900-024-00589-9 (PMC11157806; doi:10.1186/s40900-024-00589-9)
Supplement: Supplementary file 3 — Supplementary Material 3. [file 40900_2024_589_MOESM3_ESM.pdf]

Appendix 3. Example of summarized data per participant and category

Category	Participant	Summarized data
Change-making	1	Publishing in hopes that it will lead other people to publish.
	2	There is a lack of knowledge of patient perspective and therefore a need of publishing patient voice and point of view, as most research is clinical. Ultimately, the vision is integrating the care and develop a care pathway.
	3	Publishing gives some credibility. For sure /.../ I hope that this population in the end will benefit.
	4	N/A
	5	Shared their story to share that data with other patients to learn and advance understanding of their disease (...) publishing is merely a step towards the big vision.
	6	Participant has explicitly said that they want to give those who come after them, the map that that they did not have.
	7	Sharing the innovation out of obligation to ensure that as many people as possible learn... Results will create... conversations...needed to make change
	8	Prioritized getting the info out there. Sharing experiences for other patients to use.
	9	It's a good thing to get recognition...but actually what we wanted to achieve is helping ourselves and other people with illness get products they need.
	10	Publishing to get people thinking and make more aware...Desires to be slightly more recognized as a like a healthcare path.
	11	Publishing to help pharma understand what matters most to people.
	12	The publication itself was not the most important, but rather a means to a bigger goal: to have the scientific validation by addition by peers...and to help researchers or implementers being better informed.
	13	There is a whole science to be created by patients...To provide patient perspective to ensure patient opinion and accurate and reliable data regarding patient behavior.
	14	Publishing to articulate things that had not been showcased that much in research.
	15	Publishing because patient perspectives can actually advance research.
