# Supplementary material for: Why publish? An interview study exploring patient innovators’ reasons for and experiences of scientific publishing
Source: Res Involv Engagem. 2024 Jun 6;10:54. doi: 10.1186/s40900-024-00589-9 (PMC11157806; doi:10.1186/s40900-024-00589-9)
Supplement: Supplementary file 4 — Supplementary Material 4. [file 40900_2024_589_MOESM4_ESM.docx]

Appendix 4: GRIPP2-SF

| GRIPP2 Short Form | | |
| --- | --- | --- |
| Section and topic | Item | Page |
| 1. Aim | To explore patient innovators’ reasons for and experiences of authoring scientific publications about their innovations. | 5 |
| 2. Methods | Qualitative semi-structured interviews with 15 international patient innovators who had published in scientific journals. Recruitment departing from a scoping review on patient-driven innovations and snowball sampling. Data collection from June to October 2022.  Analysis of data using the Framework Method | 5-7 |
| 3. Study results | Reasons for publishing scientifically were:   - Strengthen the patient voice - Attaining recognition for the innovation - Seizing serendipitous   Experiences:   - Learning about scientific publication   - Developing skills through collaborations with professionals   - Disclosing their lived experiences - Tackling the research and publication system   - The importance of academic affiliation and funding   - Negotiations about author position and impact factor - Managing asymmetries   - Asymmetries between patient innovators and researchers   - Asymmetries between patient innovators and patient communities - Personal and innovation experience | 8-14 |
| 4. Discussion and conclusions | Research collaborations facilitate publishing:   - Consistent, continuous and meaningful collaborations defined by transparency, mutual respect, support and fair participation enhance patient innovators’ scientific skills, influence researchers’ awareness and attitudes and advance research and its relevance to end-users   Conservative research culture challenges publishing:   - Researchers and scientific journals are yet to fully acknowledge the value and contribution of patient innovators and patients’ lived experiences, and abandon box-ticking and bias - Academic affiliation requirements, funding, research legislation and regulations are perceived to cause asymmetries between patients and researchers and complicate patient involvement throughout the research process   Despite progress in increasing patient and public involvement in research and publication, our study found that continued efforts are needed to facilitate for patient innovators as well as other patient and public contributors to contribute with their experiences and expertise to the production of relevant and meaningful research. | 14-17 |
| 5. Reflection/critical perspectives | Limitations:   - Our sample may neither be representative of all patient innovators who have experiences of scientific publishing nor of patient authors who do not identify as patient innovators. - Sociodemographic factors, years of experience with scientific publishing were not assessed, which limits the transferability of our findings | 17 |
